# Differentiation of South African Game Meat Using Near-Infrared (NIR) Spectroscopy and Hierarchical Modelling

**DOI:** 10.3390/molecules25081845

**Published:** 2020-04-16

**Authors:** Kiah Edwards, Marena Manley, Louwrens C. Hoffman, Anel Beganovic, Christian G. Kirchler, Christian W. Huck, Paul J. Williams

**Affiliations:** 1 Department of Food Science, Stellenbosch University, Private Bag X1, Matieland, Stellenbosch 7602, South Africa; 17620759@sun.ac.za (K.E.); mman@sun.ac.za (M.M.); 2 Department of Animal Sciences, Stellenbosch University, Private Bag X1, Matieland, Stellenbosch 7602, South Africa; louwrens.hoffman@uq.edu.au; 3 Centre for Nutrition and Food Sciences, Queensland Alliance for Agriculture and Food Innovation (QAAFI), The University of Queensland, Health and Food Sciences Precinct, 39 Kessels Rd, Coopers Plains 4108, Australia; 4 Institute of Analytical Chemistry and Radiochemistry, CCB-Center of Chemistry and Biomedicine, Innrain 80/82, 6020 Innsbruck, Austria; Anel.Beganovic@student.uibk.ac.at (A.B.); Christian.Kirchler@uibk.ac.at (C.G.K.); Christian.W.Huck@uibk.ac.at (C.W.H.)

**Keywords:** meat fraud, game meat, near-infrared spectroscopy, spectral analysis, chemometrics, hierarchical modelling, partial least squares discriminant analysis (PLS-DA)

## Abstract

Near-infrared (NIR) spectroscopy, combined with multivariate data analysis techniques, was used to rapidly differentiate between South African game species, irrespective of the treatment (fresh or previously frozen) or the muscle type. These individual classes (fresh; previously frozen; muscle type) were also determined per species, using hierarchical modelling. Spectra were collected with a portable handheld spectrophotometer in the 908–1676-nm range. With partial least squares discriminant analysis models, we could differentiate between the species with accuracies ranging from 89.8%–93.2%. It was also possible to distinguish between fresh and previously frozen meat (90%–100% accuracy). In addition, it was possible to distinguish between ostrich muscles (100%), as well as the forequarters and hindquarters of the zebra (90.3%) and springbok (97.9%) muscles. The results confirm NIR spectroscopy’s potential as a rapid and non-destructive method for species identification, fresh and previously frozen meat differentiation, and muscle type determination.

## 1. Introduction

Environmental concern amongst consumers is leading to an increase in demand for free range and organic products, along with an increase in demand for products derived from natural production methods [1]. South African game meat can be classified as organic, as the game meat ranching is in accordance with the requirements for organic agricultural enterprises [2]. Game meat, which is considered a luxury product, is also gaining a lot of attention in an increasingly health-aware market for its natural origin and lack of antibiotics, anabolic steroids, hormones, and other additives [3].

Game meat products are purchased in various forms which include the following: frozen game meat, game biltong (jerky), dried game sausage, fresh game sausage, and fresh game meat [4]. The South African game meat industry operates as a free-market enterprise [4]; however, this can create certain problems for producers and consumers. For example, in South Africa, no standardised game meat cuts exist, and there are no quality standards in place for game [5]. This permits the legal sale of inferior-quality game meat [4]. Due to a general lack of regulation and varying carcass dressings, the chance that a species may be mislabelled or substituted is high [3].

Another concerning matter is the possibility of intentional distribution of endangered species in the market. Therefore, the identity of game meat is of mutual interest for both the meat industry and the protection of biodiversity [3].

In recent years, meat authenticity awareness increased [6], as there were incidences where meat was fraudulently mislabelled [3,7]. Typical cases involve the intentional substitution of high-value raw ingredients with inferior species or materials, the addition of non-declared proteins from several origins, or the marketing of frozen–thawed meat as fresh [8]. This type of food fraud concerns consumers in terms of economic loss, food allergies, religious compliance, and food safety [9]. Despite the potential for a sustainable game meat market, limited research was undertaken on game meat fraud.

Meat fraud was suspected to be occurring on the South African market, and a study by Cawthorn et al. [7] confirmed this suspicion with special focus on processed meats. D’Amato et al. [3], exposed the extent of unreliability of commercial labelling of game meat in South Africa. Both studies concluded that the extensive substitution and/or mislabelling of meat and wild game has important implications and is, therefore, an important authenticity issue.

Various conventional analytical methods were proposed, used, and evaluated to prevent retailers from offering fraudulent meat products [6]. Although these methods showed the potential to discriminate between species, as well as fresh and frozen-thawed meat, no single satisfactory method is yet developed. Instead, a combination of techniques is utilised to obtain reliable results. While most of the conventional techniques are able to detect low levels of adulteration with a high reliability, they are destructive, time-consuming, labour-intensive, and expensive. To date, no analytical authentication methods are yet published for the identification of meat cuts or muscle types. In the meat industry, educated staff manually differentiate between primary meat cuts through visual inspection [6]. However, the visual authentication process is more problematic when meat is cut into steaks resulting in secondary meat cuts. Muscle authentication is further complicated since the names of primary and secondary meat cuts vary between countries [6]. These factors make the establishment of objective visual criteria for authenticating specific meat cuts challenging. Thus, there is a need for cost-effective, rapid, reliable, robust, and simple alternatives. Ideally, the technique(s) should have the potential to be implemented on- or at-line in an abattoir or factory, be non-destructive, and have a high level of accuracy and reproducibility.

Near-infrared (NIR) spectroscopy meets all these requirements [10] since it is a fast, non-invasive, and cost-effective technique. Moreover, once calibrated, it is a reliable and robust method that can be installed in the manufacturing line. NIR spectroscopy is based on the interaction of NIR light with O–H, C–H, C=O, and N–H vibrations in molecules, where molecules absorb energy from light with wavelengths of 780 to 2500 nm [11]. Therefore, the spectra reveal information about the sample and its constituents, depending on how the light was either absorbed and/or scattered.

NIR spectroscopy calibrations were developed, within meat science, for the quantitative prediction of the chemical [12], physical [13], and sensory [14] characteristics of meat. NIR spectroscopy was also successfully used in discriminant analysis to recognise a specimen without the need of any chemical analysis, e.g., discrimination between different types of ground beef samples [15], the differentiation between beef breeds [16], and discrimination between fresh and frozen–thawed beef and lamb meat [17,18,19]. The technique was also used successfully to discriminate between beef and kangaroo meat [20], to discriminate between beef, pork, chicken, turkey, and lamb meat [21], and to detect and quantify adulterants in meat and minced beef [8,22], as well as to differentiate between beef cuts [23] and discriminate between three muscle types of bovine meat [16].

Therefore, NIR spectroscopy, combined with multivariate data analysis techniques, is proven to be an appropriate alternative for rapid species and muscle type identification, as well as the detection of fresh or frozen–thawed meat. However, previous studies did not combine and compare the different classes (species, muscles) and treatments (fresh, frozen–thawed) or investigate the effect thereof on the classification. Despite the broad availability of literature on NIR spectroscopy applied to meat [15], no studies were found on ostrich (*Struthio camelus*) and South African game meat species, such as zebra (*Equus quagga burchelli*) and springbok (*Antidorcas marsupialis*). Given the advances in NIR spectroscopy and chemometrics, we hypothesise that a hierarchical model will be able to differentiate between South African game species, irrespective of the meat being fresh or previously frozen, or the muscle type. The model will also be able to distinguish between these individual classes (fresh, previously frozen, muscle type) per species. The information revealed would have the potential of providing the South African game meat industry with an alternative technique to the current manual, destructive, and time-consuming methods used to detect fraud, thus contributing to the authenticity and fair trade of game meat locally and internationally.

The aim of this study was to rapidly differentiate between South African game species, irrespective of the treatment (fresh or previously frozen) or the muscle type and determine these individual classes (fresh; previously frozen; muscle type) per species, using near-infrared (NIR) spectroscopy combined with multivariate data analysis and hierarchical modelling.

## 2. Results and Discussion 

### 2.1. Species Determination

#### 2.1.1. Principal Component Analysis

**Figure 1:**
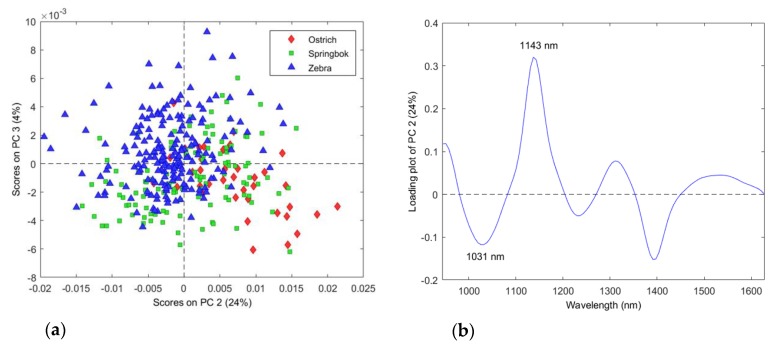
Principal component analysis (PCA; Savitzky–Golay first-derivative, second-order polynomial, seven points (SGd_1_(7) pre-processed) of species for ostrich (**red**), springbok (**green**), and zebra (**blue**) classes. Minimal class separation was observed. Scores are illustrated as (**a**) the PCA score plot of PC2 (24%) vs. PC3 (4%), and (**b**) the PCA loading line plot for PC2 with interpretable bands at 1031 and 1143 nm.

After evaluating the different pre-processing algorithms, it was found that Savitzky–Golay (first-derivative, second-order polynomial, seven points; SGd_1_(7)) was the best data pre-treatment for species identification. The score plot of PC2 (24%) vs. PC3 (4%) (Figure 1a) shows that the variation was best explained in the second component. The variance observed in the direction of PC1 (**not shown**) was attributed to intra-class separation due to the difference between fresh and frozen–thawed meat samples.

The lack of separation between the species (Figure 1a) indicates similarity in their spectral signatures; however, there are numerous aspects (e.g., physical and chemical) that could differ, leading to slight spectral differences. The reasons for the differences are most likely attributed to the fluctuation of macronutrient composition.

The correlation loadings allow identifying variables which are important to a given PC and, thus, modelled by it. Therefore, the PC2 correlation loading plot showed interpretable bands at 1031 nm (negative) and 1143 nm (positive) (Figure 1b). The band at 1031 nm (N–H stretch second overtone) is associated with protein [24], as well as meat tenderness [13]. The band at 1143 nm is associated with the C–H stretch second overtone found in aromatic groups [24], and Kamruzzaman et al. [14] reported that it corresponds to the tenderness of meat.

#### 2.1.2. Partial Least Squares Discriminant Analysis (PLS-DA)

The PLS-DA model, consisting of zebra, springbok, and ostrich, gave satisfactory discrimination results. The calibration model achieved an overall classification accuracy of 93.2% (Table 1). The ostrich class resulted in the highest classification accuracy (98.2%), followed by zebra (94.4%) and springbok (93.6%) (Table 1). Therefore, the SGd_1_(7) pre-processed data resulted in a more robust model and was less affected by variation of intrinsic parameters in meat. This suggests that the model can accurately distinguish between the three species, and this is most likely ascribed to the fact that the Savitzky–Golay first derivative eliminates noise and corrects for the baseline shift [25]. A derivative also emphasises/highlights small differences in the spectra, which are not necessarily visible in the untreated spectra. The derivatives were calculated based on a smoothing method by removing unwanted features from the data, and consequently enhancing features that are advantageous to the primary analysis.

The PLS-DA model pre-processed with SGd_1_(7) (93.2%) resulted in a slightly lower classification accuracy compared to the original model pre-processed with standard normal variate (SNV) + detrend (DT) (95.7%). Although the SNV + DT model had a higher accuracy and better individual species discrimination (Table 1), the cross-validation (CV) (94.4%) and validation (88.3%) results suggest that the model has a good predictive power for the training set, but the independent validation set was under-fitted [26]. This can also be indicative of a calibration model that was over-fitted [26]. Thus, the PLS-DA model pre-processed with SGd_1_(7) was considered to be better than the original SNV + DT model. The SGd_1_(7) model achieved lower classification (93.2%) and CV (88.9%) results, but the independent validation set achieved a higher accuracy (94.1%). This indicates that the model was more robust and not over-fitted to the training set [26]. The reason for the slight differences between these two pre-processing techniques is most likely due to the fact that both algorithms essentially do the same thing. SNV removes the scatter effects (noise) by centring and scaling each spectrum [27], and detrend reduces the baseline shift and curvature in the spectroscopic data [27]. The Savitzky–Golay first derivative does exactly the same as SNV + DT; however, the derivatives are calculated with an additional smoothing step [28].

These classification results correspond to what was reported in the literature, as previous studies mentioned that spectral derivatives have the potential to improve the classification accuracy of prediction models [22,29]. The results reported herein were also comparable to those described by McElhinney et al. [29]. The researchers suggested that the discrimination of species was based on compositional chemical differences. Cozzolino and Murray [21] reported similar results (96%) for the identification of beef, lamb, pork, and chicken meat, and a discrimination was made between the species based on intra-muscular fat, fatty acids, and moisture. Lastly, Mamani-Linares et al. [30] differentiated between beef, llama, and horse meat with accuracies between 89% and 100%. The results from the previously mentioned studies were comparable to that of the current study.

**Figure 2:**
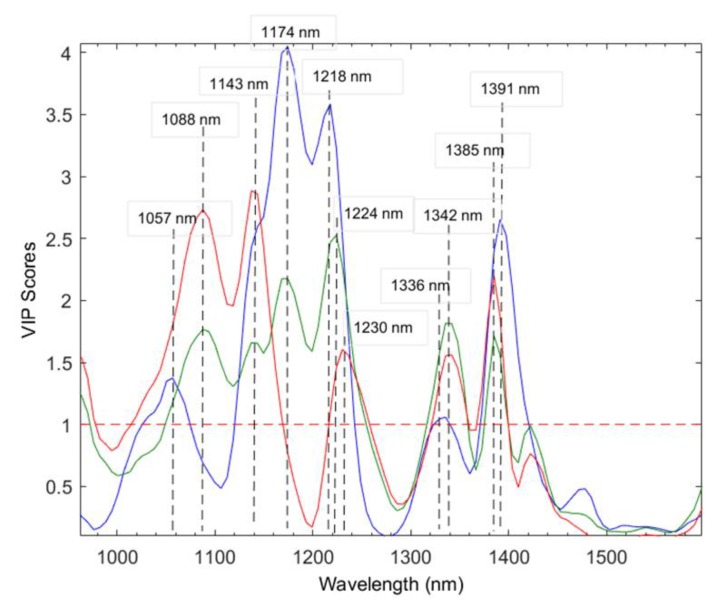
Variable importance in projection (VIP) scores for 11 waveband groups in the PLS-DA (SGd_1_(7) pre-processed) species classification model, where each VIP score above 1 represents an important spectral region.

A variable selection approach was also applied to determine which variables are contributing the most to the discrimination between the species. Variable importance in projection (VIP) scores were calculated based on the PLS-DA model, thus revealing which wavebands are the biggest drivers for the separation between the classes [31]. The VIP scores (Figure 2) illustrate the wavebands with a VIP score greater than 1, which are, thus, considered as highly influential for the separation of the classes. The 11 selected wavebands were 1057, 1088, 1143, 1174, 1218, 1224, 1230, 1336, 1342, 1385, and 1391 nm. These wavebands supported the results obtained using the PC loading values, thus indicating that the separation between the species was based on fat [32], protein [33], moisture [32], meat tenderness, and pH [13]. The advantage of using VIP scores over loading values is that it simplifies the assignment of important wavebands when numerous latent variables are calculated.

### 2.2. Fresh vs. Previously Frozen Meat Determination

#### 2.2.1. Spectral Analysis

**Figure 3:**
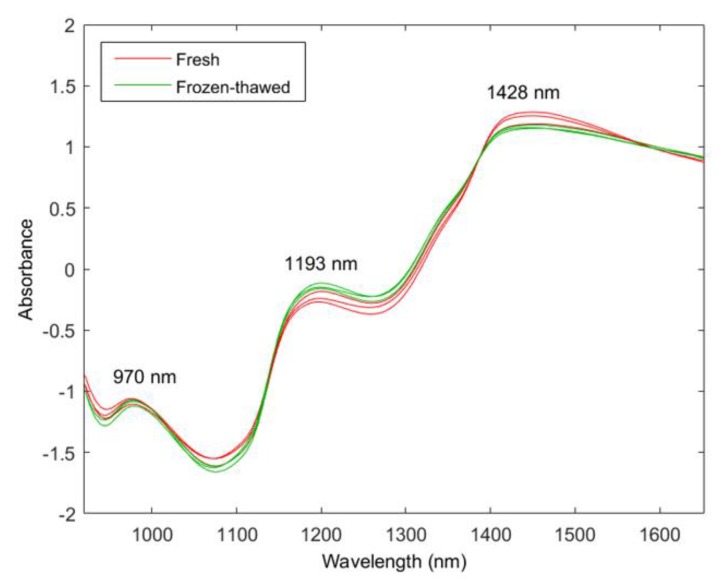
Standard normal variate (SNV) pre-processed mean spectra for fresh (**red**) and frozen–thawed (**green**) zebra, springbok, and ostrich.

The fresh and frozen–thawed mean spectra of each species (pre-processed with SNV) were computed between 920 and 1651 nm (Figure 3) to determine and compare the chemical properties. The mean spectra of the fresh and frozen–thawed samples followed a similar trend with comparable absorption bands; however, the intensity of the bands varied. The intensity differences can be attributed to the internal chemical composition. Three prominent absorption bands were exhibited at 970, 1193, and 1428 nm.

The band at 970 nm is related to the O–H second stretch overtone associated with water [21,34]. The water band of the fresh meat has a slightly higher absorption value and this could be attributed to the moisture loss of the frozen–thawed meat [35]. The 1193-nm band indicates the presence of fat (C–H stretch second overtone) as specified by Osborne et al. [24]. This band was higher in the frozen–thawed meat samples. It can be speculated that the fat concentration increased in the frozen–thawed samples due to the loss of moisture [35]. Lastly, the 1428-nm band represents the N–H stretch first overtone related to the CONH_2_ group associated with the peptide bonds in proteins [24]. This band was lower for the frozen–thawed meat samples, thus suggesting that protein denaturation occurred due to the freezing process [18]. The broad band at 1428 nm also has a contribution from moisture around 1420 and 1440 nm (O–H stretch first overtone). Thus, this band could be related to protein and moisture content [24]. This band exhibited a higher absorption for the fresh meat samples and was indicative of the expected higher moisture content, as noted by Barbin et al. [34].

#### 2.2.2. Principal Component Analysis

**Figure 4:**
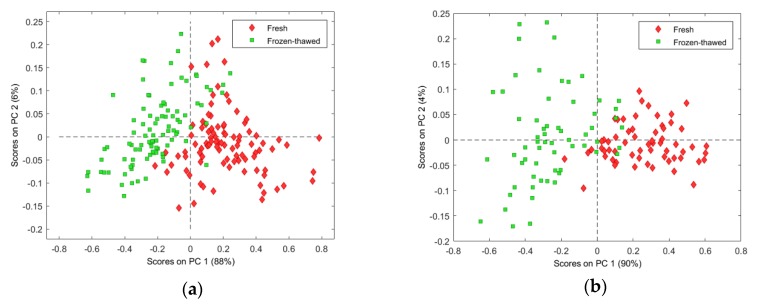

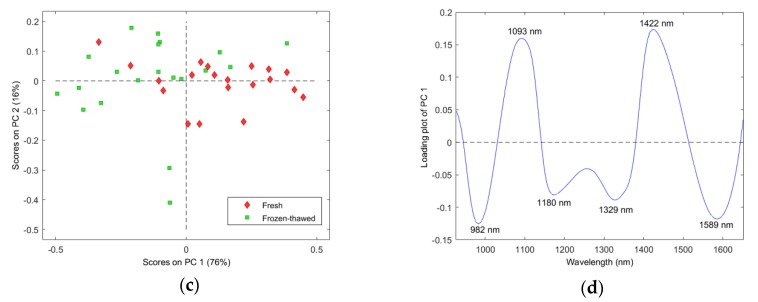
PCA analysis (SNV + detrend pre-processed) of (**a**) zebra, (**b**) springbok, and (**c**) ostrich (frozen up to one month), illustrating good separation between fresh (**red**) and frozen–thawed (**green**) classes. Scores illustrated as PCA score plots of PC1 vs. PC2; (**d**) PCA loading line plot for PC1 with interpretable bands at 982, 1093, 1180, 1329, 1422, and 1589 nm.

In Figure 4a–c, good class separation was observed between the fresh and frozen–thawed samples for all three species. The variance captured in PC1 (Figure 4a–c), for all three species, may be attributed to the inter-class separation due to the difference between fresh and frozen–thawed meat. The loading line plots of PC1 (Figure 4d) exhibited interpretable positive bands at 1093 and 1422 nm and negative bands at 982, 1180, 1329, and 1589 nm. When evaluating the positive and negative loading bands of PC1, in combination with the score plot of PC1 vs. PC2 (Figure 4a–c), the inter-class separation is mainly based on the positive spectral bands at 1093 nm, associated with pH [13], and 1422 nm, which is related to moisture [24].

Water is a major component in fresh meat and constitutes about 70%–85% [15,21]. Freezing and thawing mainly influences the water fraction of meat [36] and, due to the formation of ice crystals, causes damage to the cellular structure of the meat. As reviewed by Leygonie et al. [36], the disrupted muscle fibre structure results in a reduced water-holding capacity of meat. Thus, the main difference between fresh and frozen–thawed meat can be attributed to the loss of fluid from the meat tissue when defrosted. This decrease in moisture may cause an increase in the concentration of solutes, which consequently results in a decrease in the pH. Leygonie et al. [35] reported that the pH of previously frozen meat tends to be lower than that of fresh meat. This phenomenon is supported by the current findings, as the fresh meat samples were separated by higher loading values at 1093 nm, possibly indicative of a higher pH [13]. The current findings also illustrated that the fresh samples were separated by higher loading values at 1422 nm. We speculate that it could be indicative of an expected increased moisture content. Barbin et al. [34] reported similar results for fresh and frozen–thawed pork meat and discriminated the samples based on physical and chemical changes.

The difference between fresh and frozen–thawed meat can also be attributed to changes in the physical structure [15] caused by the formation of ice crystals [35]. Downey and Beauchêne [17] reported that freezing and thawing alters the physical structure of the meat’s surface layer, consequently changing the total reflectance spectrum. Therefore, a discrimination between fresh and frozen–thawed beef could be made based on the spectral baseline shift (Figure 2) induced by freeze–thawing [17].

#### 2.2.3. Partial Least Squares Discriminant Analysis

The PLS-DA model for springbok indicated that the fresh and frozen–thawed treatments were all correctly predicted. On the other hand, the PLS-DA model for zebra and ostrich indicated that the treatments were misclassified. The springbok model resulted in the highest classification accuracy (100%), followed by zebra (99.3%) and ostrich (90%) (Table 2).

The springbok PLS-DA models developed for the various types of pre-processing gave similar results. Although the PLS-DA models, irrespective of the pre-processing, achieved identical results, it was observed that, when using SNV + DT, the separation between classes was more distinct with little overlap. The calibration model achieved an overall classification accuracy of 100% and exhibited an excellent validation accuracy (100%), suggesting that the model could accurately distinguish between the treatments.

For the various zebra models, it was found that the PLS-DA model pre-processed with Savitzky–Golay (first-derivative, second-order polynomial, five points; SGd_1_(5)), presented the best discrimination results. This model achieved an overall classification accuracy of 99.3% (Table 2). Although the model had a calibration accuracy of 99%, the model exhibited a cross-validation (CV) of 96.5%, indicative of an effective model. Therefore, the SGd_1_(5) pre-processed data resulted in a model that was more robust. The validation accuracy also revealed that the model achieved excellent results and exhibited that the discrimination between fresh and frozen–thawed zebra meat was perfect (100%).

The overall accuracy of the ostrich PLS-DA models suggested that SGd_1_(5) pre-processing would provide the best discrimination. The PLS-DA calibration model achieved an overall classification accuracy of 90% (Table 2). Although the results exhibited calibration and validation accuracies of 90%, indicative of a model with a good fit [26], further inspection of the cross-validation results illustrated that the model achieved a slightly lower cross-validation accuracy (86.7%). This decreased CV suggests that the training set comprised samples that were more complex with regard to chemical composition. The validation set, however, suggests that the samples were simpler with less variation and, therefore, the prediction accuracy of the model was compromised. This phenomenon is one drawback of the leave-one-out cross-validation method, as the results for the sub-validations can be overly pessimistic, where “edge” samples are excluded from the calibration set [26]. These results are even more pessimistic if any of the “edge” samples are very unique in their responses.

The ostrich PLS-DA model, pre-processed with SGd_1_(5), resulted in a similar classification accuracy compared to the original model pre-processed with SNV + DT (90%). Although the SNV + DT model had similar accuracies (Table 2), the CV (83.3%) and validation (50%) results suggest that the model has a good predictive power for the training set, but not for the independent validation set. Hence, indicating that the calibration model was over-fitted [26]. The SGd_1_(5) model achieved the same classification (90%) and similar CV (86.7%) results, but the independent validation set achieved a higher accuracy (90%).

The results in the current study suggest that PLS-DA can be used to discriminate between fresh and frozen–thawed game meat samples. Ropodi et al. [19] reported similar results (93.3%) for fresh and frozen–thawed minced beef meat and discriminated the samples based on physical and chemical changes as discussed.

### 2.3. Muscle Type Determination

#### 2.3.1. Principal Component Analysis

Minimal class separation was observed in the score plots (not shown). However, there was a trend in the direction of PC2, illustrating a slight class separation between the different muscles for all three species; nevertheless, the samples exhibited an overlap between the individual muscles. Lawrie and Ledward [37] stated that the differences between muscles are very complex, making it difficult to differentiate between them. According to literature, both the dynamic (biochemical) and the static (chemical) characteristics of muscle composition are intricate [37]. The muscle variability is known to be influenced by a large number of *intrinsic* (species, breed, sex, age, anatomical location, training/exercise, inter-animal variability) and *extrinsic* factors (food, fatigue, fear, pre-slaughter manipulation, environmental conditions at slaughter).

Although the different muscles exhibit an overlap, we speculate that the slight separation could be attributed to differences in moisture [38], fat [39], protein content [24], and pH [13], as well as meat tenderness [14]. However, no correlation could be drawn between the specific factors and different muscles, within and among species. This can be attributed to the multitude of factors contributing to the separation for each species and muscle type.

#### 2.3.2. Partial Least Squares Discriminant Analysis

The overall accuracy illustrated that the ostrich model provided the best discrimination. This study found that the model for ostrich, pre-processed with SNV + detrend, could differentiate between the *gastrocnemius* (BD) and *iliofibularis* (FF) muscles with a 100% accuracy. Furthermore, the results suggest that the discrimination of the different muscle types for both zebra and springbok was less sufficient. This can be ascribed to the samples’ spectral similarities, since misclassification mostly occurred between muscles that are located near to one another anatomically. Consequently, it can be concluded that these muscles are closely related, and that the differences in their physiochemical characteristics are negligible. For this reason, it was deemed important to examine the success of a two-group discrimination model. The muscles were grouped together based on their anatomical location, and models were developed to discriminate between the forequarters (*infraspinatus* (IS), *supraspinatus* (SS)) and hindquarters (*biceps femoris* (BF), *psoas major* (fillet), *longissimus thoracis et lumborum* (LTL), *semimembranosus* (SM), *semitendinosus* (ST)), irrespective of the treatment (fresh or previously frozen).

All calibration and validation accuracies improved (Table 3); thus, the two-group discrimination improved the separation of the classes. Further examination showed that the PLS-DA models pre-processed with SNV + detrend + SGd_2_(9) for zebra (90.3%) and SNV + detrend + SGd_2_(7) for springbok (97.9%) were the best for differentiating between the muscles grouped as forequarters and hindquarters. The models pre-treated with SNV + detrend + SGd_2_(9) for zebra and SNV + detrend + SGd_2_(7) for springbok outperformed the combined pre-processing of SNV + detrend for both species, as the additional Savitzky–Golay transformation enhanced the spectral differences in protein, fat content, pH, and meat tenderness, which were predominantly the contributors for muscle separation.

These results showed that grouping samples with spectral similarities together improves the classification accuracy. McElhinney et al. [29] reported similar observations when attempting to differentiate between species. These researchers found that their model accuracies improved when chicken and turkey were combined into a single poultry class, as it was difficult to accurately discriminate between chicken and turkey meats. In our study, the results show that it was possible to differentiate between the BD and FF muscles of ostrich with 100% accuracy, irrespective of the treatment (fresh or previously frozen). In addition, it was possible to differentiate between the forequarters and hindquarters of the zebra and springbok muscles, irrespective of treatment.

### 2.4. Hierarchical Model Validation

**Figure 5:**
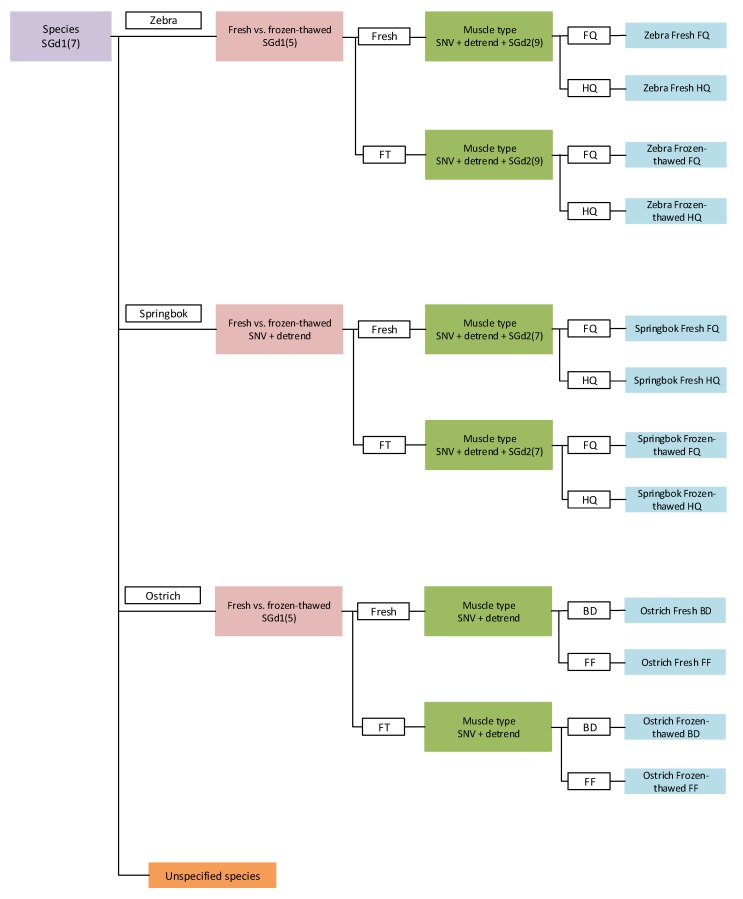
Schematic of the hierarchical model exhibiting the PLS-DA models and pre-processing used for differentiating between zebra, springbok, and ostrich, fresh and previously frozen (FT) meat, and the muscle types (forequarters (FQ), hindquarters (HQ), *gastrocnemius* (BD), and *iliofibularis* (FF)).

Due to the complexity of the data, it was not possible to classify the multiple classes (species, fresh vs. frozen–thawed, and muscle type) with a single model. To solve this problem and handle the increased detail of the data, it was divided into sub-groups with individual models. After all the models for the multiple classes were examined and the optimal models were selected, a hierarchical model (Figure 5) was constructed in which the data are organised into a tree-like structure.

The validation results for the multilevel hierarchical model (Table 4) illustrate that the model developed for species determination was able to identify most of the zebra (92.3%) and springbok (96.9%) samples, while identifying the ostrich samples perfectly (100%). Different models were then selected for each species to discriminate between the fresh and previously frozen meat, as well as to determine the different muscle types. The models for zebra and springbok could, with 100% accuracy, distinguish between the fresh and frozen–thawed meat samples. For the ostrich, on the other hand, all the frozen–thawed samples were assigned to the correct treatment (100%), while one fresh sample was incorrectly assigned to the frozen–thawed treatment, resulting in an accuracy of 80% (fresh meat determination). Lastly, the model for ostrich could differentiate between the BD and FF muscles with 100% accuracy. The springbok model achieved slightly lower accuracies, with the model able to classify all the forequarters (100%) and most of the hindquarters (93.8%). The zebra model was, to a lesser extent, able to discriminate between the forequarters and hindquarters. The model correctly classified 80.9% of the forequarters and 88.9% of the hindquarters. This hierarchical model exhibits the ability to classify multiple classes. This is preferred, as complex multiple classes can be classified using a single multilevel model, thereby simplifying the discrimination process.

## 3. Materials and Methods

### 3.1. Samples, Sampling, and Sample Preparation

This preliminary study was approved by the Animal Ethics Committee at Stellenbosch University (Ethical clearance number: SU-ACUM14-001SOP). Meat from three different South African game species (22 zebras (*Equus quagga burchelli*), 19 springboks (*Antidorcas marsupialis*), and 10 ostriches (*Struthio camelus*)) was obtained from several game and ostrich farms across the Western Cape, South Africa. The animals were randomly selected (for age and sex) and slaughtered according to standard South African procedures and regulations [40], either on-farm or in a registered abattoir, and then processed further at the Department of Animal Sciences, Stellenbosch University. From each ungulate game carcass (zebra and springbok), seven muscles (*longissimus thoracis et lumborum* (LTL), *biceps femoris* (BF), *semimembranosus* (SM), *semitendinosus* (ST), *infraspinatus* (IS), *supraspinatus* (SS), and *psoas major* (fillet)) were excised from the left side, and two muscles (*gastrocnemius* (BD) and *iliofibularis* (FF)) were excised from the left side of each ostrich carcass. Although there were only 51 animals (22 zebra, 19 springboks, 10 ostriches), due to limited sample availability, multiple muscles were investigated per animal and, due to biological variation within the muscle, each piece was seen as a sample. Therefore, altogether, there were *n* = 307 samples (154 zebras (22 × 7), 133 springboks (19 × 7), and 20 ostriches (10 × 2)). Having more animals would have been ideal for this study; however, with sustainability in mind, it was not feasible to slaughter more animals for this one project. The subcutaneous fat and visible sinews were removed from the excised muscles. The muscles were cut into 1.5–2.0-cm-thick steaks 24 h post-mortem and stored at 3 °C.

### 3.2. NIR Instrumentation and Acquisition

All the steak samples were laid down and allowed to bloom (development of pink oxymyoglobin) at ambient temperature (ca. 23 °C) for ca. 30 or 60 min, after which the near-infrared (NIR) spectra were acquired. Depending on the species, the time necessary to bloom varies. The zebra and springbok bloomed for 30 min as this was satisfactory, whereas the ostrich bloomed for 60 min since it was found to take longer to achieve optimum pink oxymyoglobin development [41]. Prior to the spectral acquisition, the surface of the meat was blotted dry with an absorbent tissue paper to remove excess moisture. All the meat samples were scanned as fresh, and the spectra were used to establish a baseline for each piece of meat per species.

Near-infrared spectra were acquired in reflectance mode with the MicroNIR OnSite (MN1700) spectrometer (Viavi Solutions Inc., Milpitas, USA) and transformed to absorbance. The illumination source comprised two integrated vacuum tungsten lamps coupled to a linear variable filter and a 128-pixel Indium Gallium Arsenide (InGaAs) photodiode array detector. Individual spectra were acquired within the spectral range of 90–1676 nm at <12.5-nm resolution with a 6.2-nm pixel-to-pixel interval and a pixel size/pitch of 30 µm × 250 µm/50 µm. All the samples were scanned in triplicate at an optimal pathlength of 3 mm (through a glass petri dish (DWK Life Sciences GmbH, Mainz, Germany)), and the measuring time per sample spectrum was 0.25–0.5 s. The NIR spectrometer was calibrated with its standard manufacturer white reference (with sapphire window). Since the glass petri dish has no influence on the spectra, and since the whole sample set of all meat species and different muscles (CAL and VAL) were measured with the same petri dish, there was no need to calibrate the petri dish. Spectra were collected while moving the NIR spectrophotometer across the sample, thus ensuring that the whole piece of meat was scanned and that most of the variation within one sample was covered. The same glass petri dish was used throughout the study and cleaned with distilled water and ethanol between sample scans.

The samples were then vacuum-packed and stored at −20 °C for one month; thereafter, the samples were thawed (at 3 °C for ca. 18 h), and the same procedure was repeated to acquire the new set of spectra for the frozen–thawed meat. Throughout this process, the samples were prepared and scanned under the same conditions (blooming time: 30 or 60 min; room temperature at measurement: 23 °C).

### 3.3. Data Analysis

The data analysis can be divided into three parts. The first part describes the procedure of the pre-treatments of the NIR spectra. This step is crucial to the success of further data analysis, as proper selection of spectral pre-treatments is capable of revealing distinct spectral features. The second part served the purpose of identifying outliers and removing them from the dataset, whereas the actual classification based on multivariate data analysis was conducted in the third part.

The data analysis was performed using The Unscrambler X Ver. 10.5 (Camo Software AS., Oslo, Norway) and the PLS_Toolbox (Eigenvector Research Inc., Wenatchee, WA, USA).

#### 3.3.1. Spectral Analysis

Prior to any pre-processing or data analysis, triplicate spectra were averaged to one spectrum per steak. In addition, spectra were reduced to a 920–1651-nm wavelength range, as noise was observed at both ends of the spectral range.

Pre-processing was performed to reduce the influence or eliminate non-relevant information from the spectra, in order to develop robust models. For consistency, the spectroscopic data was subjected to moving average smoothing (five points), which helped to reduce the noise in the data without reducing the number of variables. Several pre-processing techniques such as standard normal variate (SNV), de-trending (DT) [27], and Savitzky–Golay derivatives [28] were then evaluated in combination with different cross-validation methods (leave-one-out and venetian blinds) to determine which combination would yield the best multivariate classification model. However, it was found that the optimal pre-treatments varied among the classification targets (i.e., game species, fresh/frozen–thawed, muscle type). Therefore, the utilised spectral pre-treatments are mentioned explicitly for each model.

#### 3.3.2. Exploratory Data Analysis

Principal component analysis (PCA) [42] was applied to the mean-centred absorbance spectra. For consistency, all the PCA models were calculated with a maximum of seven principal components (PCs). Subsequently, the PCA scores plots were used to explore the data and detect potential and/or true outliers. In addition to the score plots, the influence plots were consulted for the identification of outliers. Samples identified as outliers were removed from the dataset, and the PCA models were recalculated to further explore the data, where score plots, as well as loadings and correlation loadings, were used to locate and identify clustering and important wavelengths, respectively.

#### 3.3.3. Multivariate Data Analysis

Supervised classification and discrimination models were developed to characterise the game meat samples, to differentiate between species and muscle types, and to determine whether there were differences between the fresh and previously frozen meat samples. The supervised classification and discrimination techniques evaluated were soft independent modelling of class analogy (SIMCA) [43], *K*-nearest neighbour (KNN) [44], discriminant analysis (linear (LDA), mahalanobis (MDA), quadratic (QDA)), and partial least squares discriminant analysis (PLS-DA) [45]. Although all the mentioned techniques were evaluated, only the most suitable method’s results are reported in this paper. After the data were subjected to individually optimised pre-processing techniques and outliers were removed, the following step was to split the data into a calibration and validation set using the Kennard–Stone (KS) algorithm [46].

The KS algorithm selects sample pairs with the largest Euclidean distance of *x*-vectors (predictors). Thereafter, samples are sequentially selected to maximise the Euclidean distance between *x*-vectors of the already selected and remaining samples. This is done repeatedly until the defined number of samples is attained. For each sample pair; *i* and *j*, the Euclidean distance in the *x* space is defined as Equation (1) [46].

(1) $$d_{x}\left(i,j\right)=\|x_{i}-x_{j}\|=\sqrt{\sum_{k=1}^{M}{\left(x_{ik}-x_{jk}\right)}^{2}}\;i,j\;\in \;\left[1,N\right],$$

where *x* is the array or dataset containing data to select *k* samples from, *k* is the number of samples to select, *M* is the number of variables in *x* space, *N* is the number of samples, $x_{ik}$ = is the *k*-th variable for sample *i*, and $x_{jk}$ is the *k*-th variable for sample *j*.

The number of samples selected for the calibration set comprised approximately two-thirds (70%) of the total dataset, while the remaining one-third (30%) served as the independent validation set. Upon completion of the development of calibration models, pre-processing techniques were individually optimised for each model, and their performance was evaluated. After various pre-treatments and chemometric methods were evaluated based on the cross-validation and performance measures (classification accuracy, false positive error, false negative error, sensitivity, specificity, precision, and misclassification rate), PLS-DA was identified as the most suitable classification method for the present dataset.

PLS-DA models were constructed to independently differentiate between the multiple classes. Categories were created for each of the classes, and binary dummy variables were used to indicate presence or absence during PLS-DA modelling. For example, a value of one was assigned if the spectrum belonged to the correct group or zero if it did not belong to that specific group.

The first PLS-DA model was constructed in order to discriminate between the three different game species (zebra, springbok, and ostrich), regardless of the meat being fresh or previously frozen, or of muscle type. Subsequently, for each of the different game species, PLS-DA models were developed to distinguish between fresh and previously frozen samples. Finally, for each game species and meat condition (fresh or frozen–thawed), separate PLS-DA models were constructed for the discrimination between muscle types.

The PLS-DA models were calculated using leave-one-out cross-validation, where each sample was left out of the calibration set once and subsequently predicted. Afterwards, the independent test samples were predicted to evaluate the performance of the model. The accuracy of the calibration, CV, and test set validation was assessed via the number of correctly classified samples (i.e., calibration and CV) and correctly predicted samples (i.e., test set validation). Because the study consisted of multiclass models; true positives, true negatives, false positives, and false negatives were all taken into account, even when calculating the individual class accuracies. Equation (2) shows how the accuracies were calculated.

(2) $$\mathrm{Classification}/\mathrm{prediction}\;\mathrm{accuracy}\;\left(\%\right)=\frac{\mathrm{TP}+\mathrm{TN}}{\left(\mathrm{TP}+\mathrm{TN}+\mathrm{FP}+\mathrm{FN}\right)}\;\times \;100\%,$$

where a true positive (TP) is a positive response correctly classified as a positive response, a true negatives (TN) is a negative response correctly classified as a negative response, a false positive (FP) is a negative response incorrectly classified as a positive response, and a false negative (FN) is a positive response incorrectly classified as a negative response.

Additionally, a multilevel hierarchical model was constructed by selecting the general categories (species). Afterwards, sub-models were used to further divide (e.g., fresh vs. frozen–thawed and muscle type) those general categories using increasingly specific classification models. This multilevel hierarchical model was then used to predict the independent test set.

## 4. Conclusions

Meat authenticity and traceability are important issues, as incidences regarding meat adulteration and fraud are becoming more sophisticated and mainstream. Our results in this preliminary study show that NIR spectroscopy can be used as a rapid and non-destructive method for game meat authentication. The use of multilevel hierarchical modelling enables one to simultaneously classify multiple classes, without using individual models for each class determination. This approach provides a holistic viewpoint of the meat samples’ characteristics and highlights the fact that different types of models (data pre-treatment and number of latent variables) may be suitable for each class discrimination step, thus confirming that it is not always possible to use a single model (same data pre-treatment and number of latent variables) to classify complex data with multiple classes. Since conventional NIR spectroscopy only provides an averaged spectrum per sample, this work should be extended to NIR hyperspectral imaging.

However, despite the promising results of this study, more samples are required to establish reliable and robust multivariate models and successfully manage the transfer of these models to the industry. In addition, future studies should investigate the freezing, thawing, and scanning conditions, expand the spectral database by including more game species from different regions and seasons, and investigate the classification potential of game meat emulsions containing different adulterants at varying concentrations.

## Figures and Tables

**Table 1:** An overview of the accuracies of the partial least squares discriminant analysis (PLS-DA) models with standard normal variate (SNV) + detrend and Savitzky–Golay (first-derivative, second-order polynomial, seven points) pre-processing applied to distinguish between species.

Game Species	Pre-Processing		Calibration					Validation		
		No of LVs ^1^	*nT* ^6^	*nC* ^7^	Classification Accuracy (%)	*nC* ^7^	CV ^2^ (%)	*nT* ^6^	*nP* ^8^	Prediction Accuracy (%)
Overall	SNV ^3^ + DT ^4^	8	233	223	95.7	220	94.4	120	106	88.3
	SGd_1_(7) ^5^	7	235	219	93.2	209	88.9	118	111	94.1
Zebra	SNV ^3^ + DT ^4^	8	120	118	98.2	118	97.4	72	70	92.2
	SGd_1_(7) ^5^	7	111	103	94.4	99	91.3	80	74	94.9
Springbok	SNV ^3^ + DT ^4^	8	81	74	95.7	72	94.4	43	31	88.3
	SGd_1_(7) ^5^	7	95	88	93.6	83	89.3	29	28	94.1
Ostrich	SNV ^3^ + DT ^4^	8	32	31	97.4	30	96.9	5	5	95.5
	SGd_1_(7) ^5^	7	29	28	98.2	27	96.8	9	9	99.1

^1^ (LVs) latent variables; ^2^ (CV) cross-validation; ^3^ (SNV) standard normal variate; ^4^ (DT) detrend; ^5^ (SGd_1_(7)) Savitzky–Golay (first-derivative, second-order polynomial, seven points); ^6^ (*nT*) total number of samples; ^7^ (*nC*) correctly classified samples; ^8^ (*nP*) correctly predicted samples.

**Table 2:** An overview of the accuracies of the PLS-DA models with various pre-processing techniques applied to distinguish between fresh or previously frozen meat.

Game Species	Pre-Processing		Calibration					Validation		
		No of LVs ^1^	*nT* ^7^	*nC* ^8^	Classification Accuracy (%)	*nC* ^8^	CV ^2^ (%)	*nT* ^7^	*nP* ^9^	Prediction Accuracy (%)
Zebra	SNV ^3^ + DT ^4^	3	144	137	95.1	134	93.1	48	48	100
	SGd_1_(5) ^5^	5	144	143	99.3	139	96.5	48	48	100
Springbok	SNV ^3^ + DT ^4^	2	93	93	100	93	100	31	31	100
	SNV ^3^ + DT ^4^ + SGd_2_(7) ^6^	2	93	93	100	90	96.7	31	31	100
Ostrich	SNV ^3^ + DT ^4^	1	30	27	90	25	88.3	10	5	50
	SGd_1_(5) ^5^	3	30	27	90	26	86.7	10	9	90

^1^ (LVs) latent variables; ^2^ (CV) cross-validation; ^3^ (SNV) standard normal variate; ^4^ (DT) detrend; ^5^ (SGd_1_(5)) Savitzky–Golay (first-derivative, second-order polynomial, five points); ^6^ (SGd_2_(7)) Savitzky–Golay (second-derivative, second-order polynomial, seven points); ^7^ (*nT*) total number of samples; ^8^ (*nC*) correctly classified samples; ^9^ (*nP*) correctly predicted samples.

**Table 3:** An overview of the accuracies of the PLS-DA models with various pre-processing techniques applied to distinguish between the muscle types.

Game Species	Pre-Processing		Calibration					Validation		
		No of LVs ^1^	*nT* ^7^	*nC* ^8^	Classification Accuracy (%)	*nC* ^8^	CV ^2^ (%)	*nT* ^7^	*nP* ^9^	Prediction Accuracy (%)
Zebra	SNV ^3^ + DT ^4^	4	144	118	81.9	116	80.6	48	39	81.3
	SNV ^3^ + DT ^4^ + SGd_2_(9) ^6^	5	144	130	90.3	120	83.3	48	41	85.4
Springbok	SNV ^3^ + DT ^4^	3	93	78	83.9	75	80.7	31	19	61.3
	SNV ^3^ + DT ^4^ + SGd_2_(7) ^5^	5	93	91	97.9	83	89.3	31	30	96.8
Ostrich	SNV ^3^ + DT ^4^	5	30	30	100	30	100	10	10	100
	SGd_2_(7) ^5^	5	30	30	100	29	96.7	10	10	100

^1^ (LVs) latent variables; ^2^ (CV) cross-validation; ^3^ (SNV) standard normal variate; ^4^ (DT) detrend; ^5^ (SGd_2_(7)) Savitzky–Golay (second-derivative, second-order polynomial, seven points); ^6^ (SGd_2_(9)) Savitzky–Golay (second-derivative, second-order polynomial, nine points); ^7^ (*nT*) total number of samples; ^8^ (*nC*) correctly classified samples; ^9^ (*nP*) correctly predicted samples.

**Table 4:** An overview of the accuracies of the PLS-DA models with various pre-processing techniques applied to distinguish between the muscle types.

Data Pre-Treatment/LVs ^1^		Correct Prediction		Data Pre-Treatment/LVs ^1^		Correct Prediction		Data Pre-Treatment/LVs ^1^		Correct Prediction	
Species Classification				Fresh vs. Frozen-Thawed				Muscle Type			
**SGd_1_(7)**^7^/**8 LVs**^1^	***nT*** ^10^	***nP*** ^11^	%	**SGd_1_(5)**^6^/**5 LVs**^1^	***nT*** ^10^	***nP*** ^11^	%	**SNV**^2^ + **DT** ^3^ + **SGd_2_(9)** ^5^/**6 LVs** ^1^	***nT*** ^10^	***nP*** ^11^	%
**Zebra**	52	48	92.3	**Fresh** **Frozen-thawed**	21 27	21 27	100 100	**Forequarters** **Hindquarters**	21 27	17 24	80.9 88.9
**Springbok**	***nT*** ^10^	***nP*** ^11^	%	**SNV**^2^ + **DT** ^3^/**2 LVs** ^1^	***nT*** ^10^	***nP*** ^11^	%	**SNV**^2^ + **DT** ^3^ + **SGd_2_(7)** ^4^/**5 LVs** ^1^	***nT*** ^10^	***nP*** ^11^	%
	32	31	96.9	**Fresh** **Frozen-thawed**	18 13	18 13	100 100	**Forequarters** **Hindquarters**	15 16	15 15	100 93.8
**Ostrich**	***nT*** ^10^	***nP*** ^11^	%	**SGd_1_(5)**^6^/**3 LVs**^1^	***nT*** ^10^	***nP*** ^11^	%	**SNV**^2^ + **DT** ^3^/**5 LVs** ^1^	***nT*** ^10^	***nP*** ^11^	%
	9	9	100	**Fresh** **Frozen-thawed**	5 4	4 4	80 100	**BD** ^8^ **FF** ^9^	4 4	4 4	100 100

^1^ (LVs) latent variables; ^2^ (SNV) standard normal variate; ^3^ (DT) detrend; ^4^ (SGd_2_(7)) Savitzky–Golay (second-derivative, second-order polynomial, seven points); ^5^ (SGd_2_(9)) Savitzky–Golay (second-derivative, second-order polynomial, nine points); ^6^ (SGd_1_(5)) Savitzky–Golay (first-derivative, second-order polynomial, five points); ^7^ (SGd_1_(7)) Savitzky–Golay (first-derivative, second-order polynomial, seven points); ^8^ (BD) *gastrocnemius*; ^9^ (FF) *iliofibularis*; ^10^ (*nT*) total number of samples; ^11^ (*nP*) correctly predicted samples.
